# Triggers for the onset and recurrence of psoriasis: a review and update

**DOI:** 10.1186/s12964-023-01381-0

**Published:** 2024-02-12

**Authors:** Suwen Liu, Mengwen He, Jian Jiang, Xiaoru Duan, Bao Chai, Jingyu Zhang, Qingxiao Tao, Hongxiang Chen

**Affiliations:** 1 Department of Dermatology, Union Hospital, Tongji Medical College, Huazhong University of Science and Technology, Wuhan, 430022 China; 2 Department of Dermatology, Huazhong University of Science and Technology Union Shenzhen Hospital, Shenzhen, 518052 China; 3 Department of Rheumatology and Immunology, Union Hospital, Tongji Medical College, Huazhong University of Science and Technology, Wuhan, 430022 China; 4 Department of Dermatology, The 6th Affiliated Hospital of Shenzhen University Medical School, Shenzhen, 518052 China

**Keywords:** Psoriasis, Infections, Microbiota dysbiosis, Dysregulated lipid metabolism, Psychological stress, Environmental triggers

## Abstract

Psoriasis is an immune-mediated inflammatory skin disease, involving a complex interplay between genetic and environmental factors. Previous studies have demonstrated that genetic factors play a major role in the pathogenesis of psoriasis. However, non-genetic factors are also necessary to trigger the onset and recurrence of psoriasis in genetically predisposed individuals, which include infections, microbiota dysbiosis of the skin and gut, dysregulated lipid metabolism, dysregulated sex hormones, and mental illness. Psoriasis can also be induced by other environmental triggers, such as skin trauma, unhealthy lifestyles, and medications. Understanding how these triggers play a role in the onset and recurrence of psoriasis provides insights into psoriasis pathogenesis, as well as better clinical administration. In this review, we summarize the triggers for the onset and recurrence of psoriasis and update the current evidence on the underlying mechanism of how these factors elicit the disease.

Video Abstract

**Supplementary Information:**

The online version contains supplementary material available at 10.1186/s12964-023-01381-0.

## Background

**Fig. 1:**
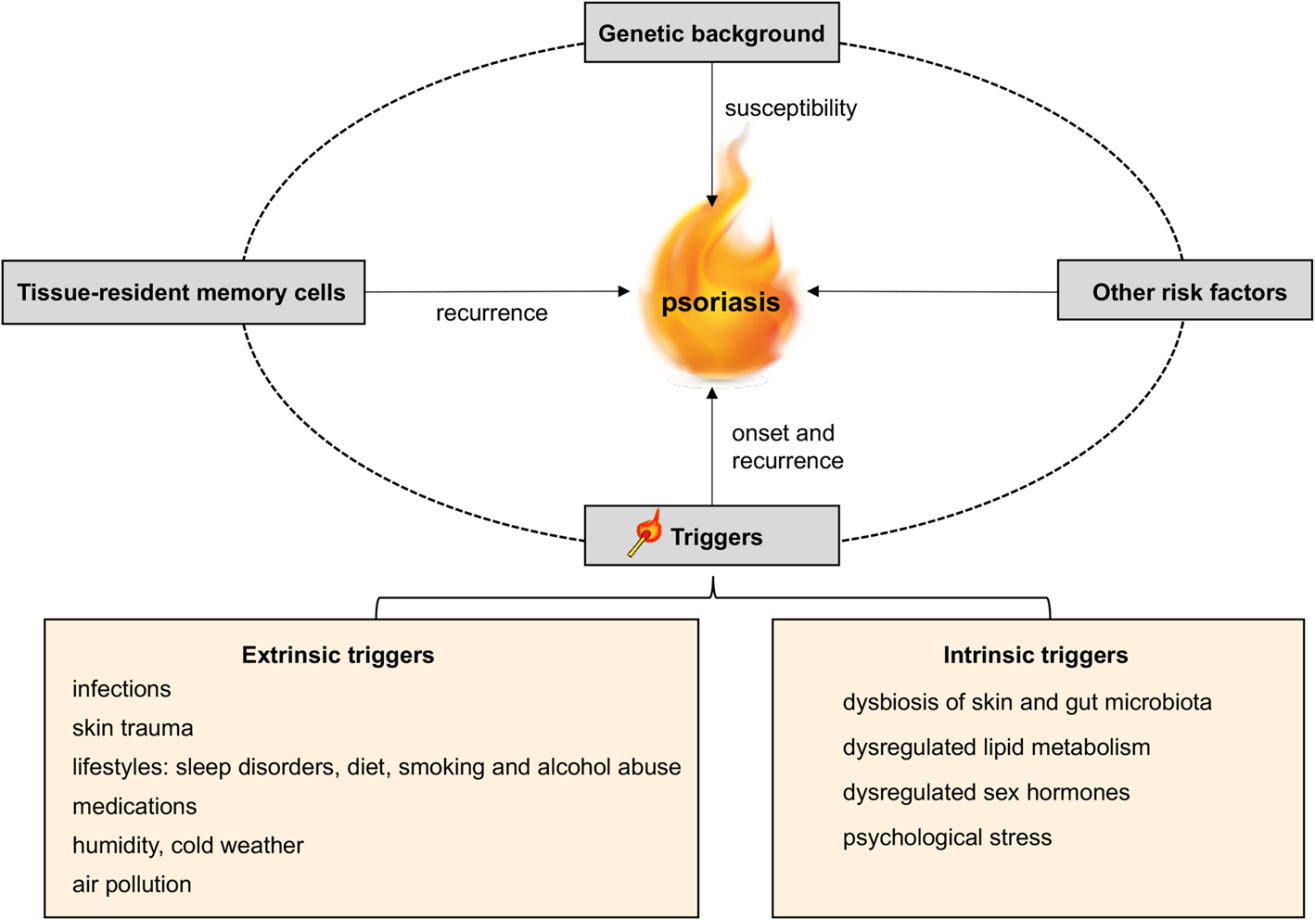
The extrinsic and intrinsic triggers for the onset and recurrence of psoriasis. Psoriasis can be induced by these triggers under a genetic predisposition. The recurrence of psoriasis also involves the role of tissue-resident memory cells

Psoriasis is a T-cell-mediated chronic inflammatory skin disease, which is characterized by excessive proliferation of keratinocytes (KCs) as well as redness caused by dilated dermal blood vessels and infiltration of immune cells [1]. Immune-related cells including dendritic cells (DCs) and T helper (Th) 17 cells, along with Toll-like receptors(TLRs) and cytokines such as interferon (IFN)-α, tumor necrosis factor (TNF)-α, IFN-γ, Interleukin(IL)-12, IL-22, IL-23, and IL-17, are responsible for the pathogenesis of psoriasis [2]. However, the exact etiology and pathogenesis are awaited to be elucidated [3]. Genome-wide association studies (GWAS) have identified more than 60 psoriasis susceptibility loci, which largely contribute to a better understanding of disease mechanisms and related pathways [1]. Still, it appears that the loss of immunological tolerance is a result of the close interplay between genetic factors and environmental triggers [4]. Therefore, identifying these specific triggers and unraveling their mechanism are crucial for the development of new therapies or interventions for psoriasis. This review is focused on the triggers for psoriasis, including extrinsic and intrinsic risk factors. The former include infections [5], skin trauma [6], lifestyles [7–9], mediation [10–15], humidity [16], cold weather [16], and air pollution [17]; while the latter include microbiota dysbiosis [18, 19], stress [20], dysregulated lipid metabolism [21], and dysregulated sex hormones [22] (Fig. 1).

It is worth noting that these risk factors have the potential to trigger both the onset and the recurrence of psoriasis, especially when these triggers persist as long-term stress events. Beyond the relevant triggers that will be discussed in this review, the underlying mechanisms of psoriasis recurrence at the originally affected sites are intricately linked to tissue-resident memory cells (TRM) in the skin [23]. In psoriatic plaques that have resolved after treatment, CD8 + TRM are retained within the epidermis, while CD4 + TRM the dermis. They all derived from their respective circulating memory cells [24]. When stimulated by triggering events, DCs and Langerhans cells secrete IL-23, which interacts with IL-23R on the surface of TRM, particularly IL-17‒producing CD49a–CD103 + CD8 + TRM, resulting in the reinitiating of inflammatory loops in the psoriatic skin [25]. Furthermore, the concept of a "molecular scar" within the epidermis of resolved lesions has been proposed, which is characterized by the inability of a specific set of genes, including those coding proinflammatory molecules IL-12, IFN-induced guanosine triphosphate binding protein Mx1 (MX1), IL-22, IL-17 and IFN‐γ, to revert to their normal expression levels [26].

## Infections

**Fig. 2:**
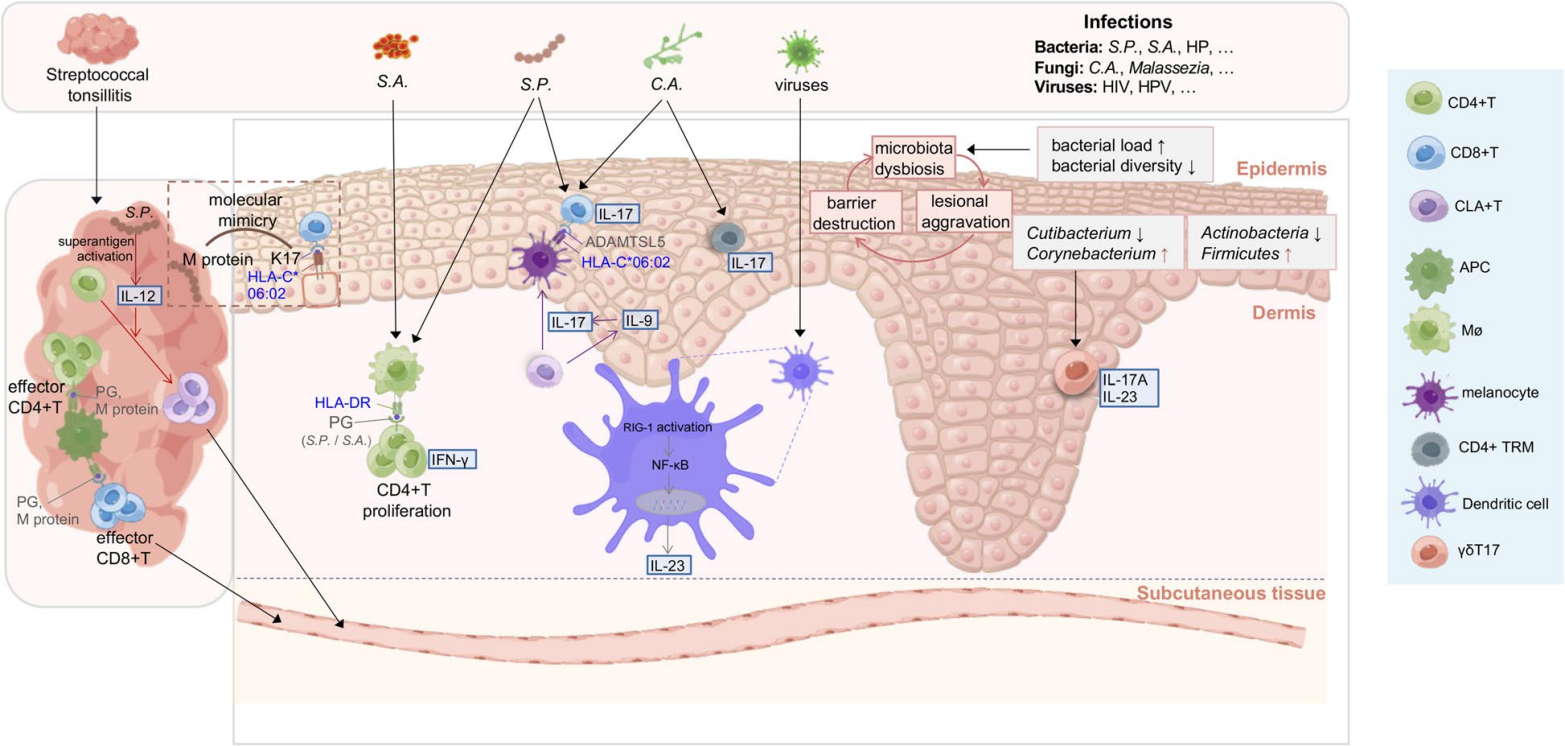
Potential role of infections and dysbiosis of skin microbiota in triggering psoriasis (by Figdraw, www.figdraw.com). Streptococcal tonsillitis may trigger psoriasis through various mechanisms: **a**) M protein of *Streptococcus pyogenes (S.P.)* mimicking human K17, **b**) superantigens of *S.P.* activating the release of IL-12 and then promoting the expression of skin-homing CLA in CD4 + T cells, and **c**) adjuvant effects of streptococcal PG. Regarding to other infections, macrophages (Møs) presents PG of *Staphylococcus aureus (S.A.)* and *S.P.* by HLA-DR to CD4 + T cells, leading to the proliferation of CD4 + T and the production of IFN-γ. *S.P* and *Candida albicans (C.A.)* colonizing in psoriatic lesions induce the migration of IL-17–producing CLA + T cells to the skin and expression of psoriasis autoantigens like ADAMTSL5, which is recognized by autoreactive CD8 + T cells in epidermis. The *C. A.* infection also induces IL-17 production by CD4 + TRM. Virus infection activates RIG-I antiviral signaling in CD11c + DCs and induces the IL-23 expression through NF-κB. A vicious cycle starting from barrier destruction to microbiota disturbance, then to lesion aggravation promotes the formation of psoriatic inflammation. The high level of *Corynebacterium* in psoriatic skin lesions induces an intense IL-17 and IL-23-dependent response of γδT17

The theory that psoriasis is infection-provoked has been widely concerned. Various microorganisms have been reported as the triggers of psoriasis, and many efforts have been devoted to the clarification of the mechanisms (Fig. 2). The pathogens that provoke psoriasis are summarized in Table 1.

**Table 1:** List of pathogens associated with the onset or recurrence of psoriasis

Pathogens	Routes of Infections	References
**Bacteria**		
*Streptococci pyogenes*	tonsil	Thorleifsdottir et al. 2016 [27]
*Staphylococcus aureus*	skin, tonsil	Ng et al. 2017 [28]
*Mycoplasma faucium*	oral cavity	Arabatzis et al. 2022 [29]
*Porphyromonas gingivalis*	oral cavity	Moen et al. 2006 [30], Cheng et al. 2016 [31]
*Aggregatibacter actinomycetemcomitans*	oral cavity	Moen et al. 2006 [30]
*Helicobacter pylori*	digestive tract	Yu et al. 2019 [32]
*Chlamydophila psittaci*	respiratory transmission	Stinco et al. 2012 [33]
**Viruses**		
Human immunodeficiency virus	maternal–fetal, sexual, transfusion-related transmission	Morar et al. 2010 [34]
Human papillomavirus	skin or mucous membranes	Chen et al. 2020 [35]
Hepatitis C virus	blood transmission	Chun et al. 2017 [36]
Varicella zoster virus	skin	Garg et al. 2012 [37]
Epstein-Barr virus	oral transmission	Jiyad et al. 2015 [38]
Parvovirus B19	respiratory transmission	Yazici et al. 2006 [39]
Cytomegalovirus	humoral transmission	Weitz et al. 2011 [40] Yoneda et al. 2012 [41]
Zika virus	arthropod-borne, maternal–fetal, sexual, and transfusion-related transmission	Paniz et al. 2018 [42]
Coxsackie B	digestive and respiratory transmission	Korzhova et al. 2001 [43]
Human endogenous retrovirus	skin	Molès et al. 2005 [44]
Chikungunya	arthropod-borne transmission	Seetharam et al. 2011 [45]
COVID-19	respiratory transmission	Kutlu et al. 2020 [46]
**Fungi**		
*Candida albicans*	skin	de Jesús-Gil C et al. 2021 [47], Park et al. 2018 [48]
*Malassezia*	skin	Rudramurthy et al. 2014 [49]

### *Streptococcus pyogenes*

It has been acknowledged that tonsillar infections caused by *S. pyogenes* can trigger or exacerbate psoriatic skin lesions in both plaque and guttate psoriasis [27, 50]. Researchers have linked streptococcal throat infections to psoriasis through genetic association studies, suggesting the recognized psoriasis risk allele HLA-C*06:02 as a risk factor for streptococcal tonsillitis and the imputed psoriasis risk haplotype HLA-C*06:02/HLA-B*57:01 as the strongest risk for tonsillitis [51, 52]. A clinical cohort study also reported that pediatric psoriasis aging from 10 to 11 was strongly associated with recurrent tonsillitis [53]. The same T cell clones were observed in psoriatic patients’ skin and tonsillar tissue, proposing the production of pathogenic T cells within the tonsils in post-streptococcal disorders [54]. Accordingly, tonsillectomy has been recommended as an intervention to resolve psoriasis, which can decrease the number of circulating T cells [55, 56]. Still, long-term follow-up should be conducted to verify the indication and long-lasting benefit of tonsillectomy [57]. Meanwhile, there is no solid evidence of the effectiveness of anti-streptococcal interventions [58]. Interestingly, the perianal streptococcal infection can trigger guttate psoriasis as well, but it appears less common than throat infections [59].

A classical explanation for the pathogenetic links between *S. pyogenes* throat infections and psoriasis is molecular mimicry. CD8 + T cells recognize epitopes shared by streptococcal M proteins and human keratin 17 (K17) in psoriatic patients, and K17 can become the self-antigen and target of the CD8 + T cells infiltrating the psoriatic skin lesions in an HLA-C*06:02–restricted pattern [60, 61].

The interaction of skin-seeking cutaneous lymphocyte-associated T cells (CLA + T cells) with *S. pyogenes* provides novel concepts to understand the immunopathogenesis of psoriasis [62]. Through stimulation of the IL-12 production pathway, *S. pyogenes* superantigens induce the expression of skin-specific homing receptors (the CLA antigen) on T cells and promote the migration of CLA + T cells to the skin [63]. Moreover, a high Th17 response has been observed in the cultures of CLA + T cells and epidermal cells from HLA-C*06:02–associated psoriatic patients with streptococcal tonsillitis [64]. *S. pyogenes* can induce IL-17 production in circulating CLA + T cells both in plaque and guttate psoriasis, which further induces psoriasis autoantigens (such as ADAMTS-like protein 5 and LL-37) after the CLA + T cells migrate to the skin [65]. In a psoriatic model in vitro, extracts of *S. pyogenes* induced the CLA + T cells to produce IL-9, which upregulates IL-17A production [66].

*S. pyogenes* peptidoglycan (PG) is also responsible for T cell activation in psoriasis. PG-containing macrophages are in close contact with PG-specific CD4 + T cells in psoriatic lesions, then the PG-specific CD4 + T cells proliferate and produce IFN-γ in an HLA-DR allele-restricted manner [67]. Additionally, the altered innate recognition of PG enhances the responses of T cells to *S. pyogenes* and induces psoriasis [68].

### *Staphylococcus aureus*

*S. aureus* colonizes psoriatic skin lesions and nares in approximately 60% of psoriasis patients, while the colonization is observed in 5% to 30% of healthy individuals [10]. *S. aureus* was isolated from the throats of 11 of 22 psoriasis patients [69]. A study revealed an increase in inflammatory skin response to superantigen toxins in psoriatic subjects and an increased level of TNF-α mRNA in the psoriatic epidermis compared to healthy controls. However, the selective expansion of T cells expressing specific T cell receptor Vβ, a hallmark of superantigen stimulation, was not seen in psoriatic lesions. This T-cell-independent response might be explained by the higher expression of HLA-DR in KCs that enhanced inflammatory skin responses to superantigens [70]. Additionally, the severity of psoriasis was shown to significantly correlate with the production of staphylococcal enterotoxin, though mechanisms underlying this phenomenon remain unclear [71].

### Commensal bacteria of the oral cavity

During periodontitis, the oral microbiota may affect the development and exacerbation of psoriasis [72]. A meta-analysis involving 13 studies has shown that the risk of developing psoriasis was higher in patients with periodontitis than in the control group [73]. One patient with initial guttate and later plaque psoriasis was cutaneously infected with *Mycoplasma faucium*, an oral *Tenericutes* species, which presented in the KCs of psoriatic stratum spinosum and extracellularly in the upper dermis of the psoriatic lesions [29]. Higher varieties and concentrations of oral bacterial (*Porphyromonas gingivalis* and *Prevotella nigrescens*) DNAs were also found in serum and synovial fluid of psoriatic arthritis (PsA) patients compared to controls (osteoarthritis) [30].

*P. gingivalis* and *Aggregatibacter actinomycetemcomitans*, pathogens associated with perodontitis, can activate human CD14 + monocytes to enhance Th17 differentiation and IL-17 production in vitro. *P. gingivalis* proteases can enhance Th17 lineage responses by degrading other crucial cytokines like IL-12, and myeloid antigen-presenting cells (APCs) are triggered to produce Th17-related cytokines IL-1β, IL-6, and IL-23 [74]. However, compared to healthy subjects, the frequency of IL-17 + cells was increased in patients with periodontitis in gingival tissue, not in peripheral blood [31].

### Viruses

The skin inflammation in psoriasis can be triggered by the viral infection through the dysregulation of the antiviral immune response of hosts. Retinoic acid inducible-gene I (RIG-I) is the main cytoplasmic sensor of viruses. By activating RIG-I antiviral signaling, the infection of viruses can trigger the expression of IL-23 in the CD11c + DCs in genetically predisposed individuals, thereby leading to the development of psoriasis [75].

Human Immunodeficiency Virus (HIV)-infected patients have higher standardized incidence rates for psoriasis as compared to the general population [76]. HIV can directly trigger psoriasis as a source of superantigens or as a costimulatory factor in antigen presentation [34], and more IFN-γ is produced by activated CD8 + T cells during HIV infections [77]. The neuropeptide substance P can be released from HIV-infected immune cells and then modulates inflammatory and immune responses and stimulates the proliferation of KCs [78]. Human papillomavirus (HPV) is noted to be associated with psoriasis as well. A nationwide population-based cohort study that enrolled 66,274 patients with HPV infections revealed a higher prevalence of psoriasis after HPV infections [35].

Severe Acute Respiratory Syndrome Coronavirus 2 (SARS-CoV2) was also proposed to be responsible for the exacerbation of psoriasis [5]. An enhanced level of inflammatory cytokines was observed in the plasma of SARS-CoV2-infected patients, and the concentrations of granulocyte-colony stimulating factor and TNF-α were associated with disease severity [79]. Additionally, some patients who received COVID-19 vaccines were reported to suffer from the exacerbation of chronic immune-mediated dermatoses like psoriasis, but the cutaneous reactions were generally mild and self-limiting [80, 81].

Nucleotide-binding domain and leucine-rich repeat pyrin domain-containing protein 1 (NLRP1) was one of the identified inflammasome-forming pattern recognition receptors (PRRs), by which the innate immune system can detect pathogens. Long double-stranded RNA (dsRNA) generated in the course of infections of positive-strand RNA viruses, e.g., Semliki Forest virus, can bind and activate NLRP1 inflammasome in human keratinocytes [82]. NLPR1 inflammasome has been implied in prompting the onset of psoriasis, either by increasing the susceptibility to psoriasis or by dysregulated release of pro-inflammatory cytokines including IL-1β and IL-18 [83–85]. Very similarly, NLRP1 has the capacity to sense bacterial pathogen exotoxin, such as exotoxin A secreted by *Pseudomonas aeruginosa* and diphtheria toxin by *Corynebacterium diphtheriae*, and induce cell death and IL-1β/ IL-18 secretion [86].

### Other pathogens: fungal microbiota, *helicobacter pylori*, and so on

Diverse fungi in psoriatic skin have been identified to activate psoriasis through the innate immune system in genetically predisposed individuals [87]. As a typical example, *Candida albicans* has been frequently found in intertriginous psoriasis. Superantigens derived from microbes such as *C. albicans* might lead to the exacerbation of psoriasis in infected patients [88]. Exposure to *C. albicans* can also trigger a clinically relevant response to IL-17 in psoriatic skin [47]. Psoriatic CLA + T cells/ epidermal cells co-cultures responded to *C. albicans* extract by increasing the production of IL-9, IL-17A, and IFN-γ [66]. Moreover, cutaneous *C. albicans* infection induced recurrent psoriasis through IL-17-producing CD4 + TRM. In a mouse model, the CD4 + TRM become the main source of IL-17 after 30 days of infection. Other than *C. albicans*, *Malassezia organisms* may be implicated in the exacerbation of scalp psoriasis [49].

Compared to the control groups, *H. pylori* infections were significantly increased among moderate and severe psoriatic patients, but not among mild psoriatic patients [32]. In psoriasis patients with *H. pylori* infections, the Psoriasis Area and Severity Index (PASI) scores were higher [32, 89], so were the mucosal levels of psoriasis-associated cytokines IL-1β, IL-6, IL-8, and TNF-α [90]. However, a finding in 2015 argued that there was no increased prevalence of *H. pylori* in psoriasis individuals than in healthy controls [91].

## Dysbiosis of skin and gut microbiota

Currently, much research has been devoted to the role of the human microbiome in the pathogenesis of psoriasis, especially the relationship between cutaneous and intestinal microbiomes, known as the “gut-skin axis” [18].

Several researchers have speculated that psoriasis may be closely associated with the dysbiosis of skin microbiota in the host (Fig. 2). A higher bacterial load but lower bacterial diversity in lesional psoriatic skin were recently revealed, compared to non-lesional skin and controls [92, 93]. *Firmicutes* and *Actinobacteria* are respectively the most common bacterial phylum in psoriatic patients and healthy controls [94], and an increased *Firmicutes* and a corresponding decrease in *Actinobacteria* were significant in lesional skin [95]. However, another study reported an increase in both *Actinobacteria* and *Firmicutes* in psoriasis lesions [96]. This discrepancy may be due to the variety of sampling methods, skin sites, medications, and analytical methodologies [97]. According to new evidence, compared to unaffected and healthy skin, psoriatic lesions have higher concentrations of *Corynebacterium* and lower concentrations of *Cutibacterium* [92]. *Corynebacterium* abundance was correlated with disease severity [92] and most species of *Corynebacterium* induce an intense IL-23-dependent response in mouse skin [98]. After smearing *Corynebacterium pseudodiphtheriticum* on mouse skin, the cutaneous IL-1β protein level and γδT17 cells in the dermis were increased [99]. Moreover, the psoriasis ear skin showed an overrepresentation of *staphylococci* [100]. A lower abundance of *Staphylococcus epidermidis* and *Propionibacterium acnes* may promote *S. aureus* colonization in psoriasis, which can stimulate Th17 polarization and trigger IL-17-mediated skin inflammation in a mice model [101].

A cycle from barrier destruction to microbiota disturbance, then to lesion aggravation was proposed to explain the pathogenesis of psoriasis [92, 102]. Mice with epidermal barrier defects have an increased bacterial load and antimicrobial peptides (AMPs) expression. The psoriasis-like phenotype in the mice could be relieved by reducing bacterial load on the skin after applying topical antibiotics, along with the decrease of IL-17 and IL-22 production [103].

**Fig. 3:**
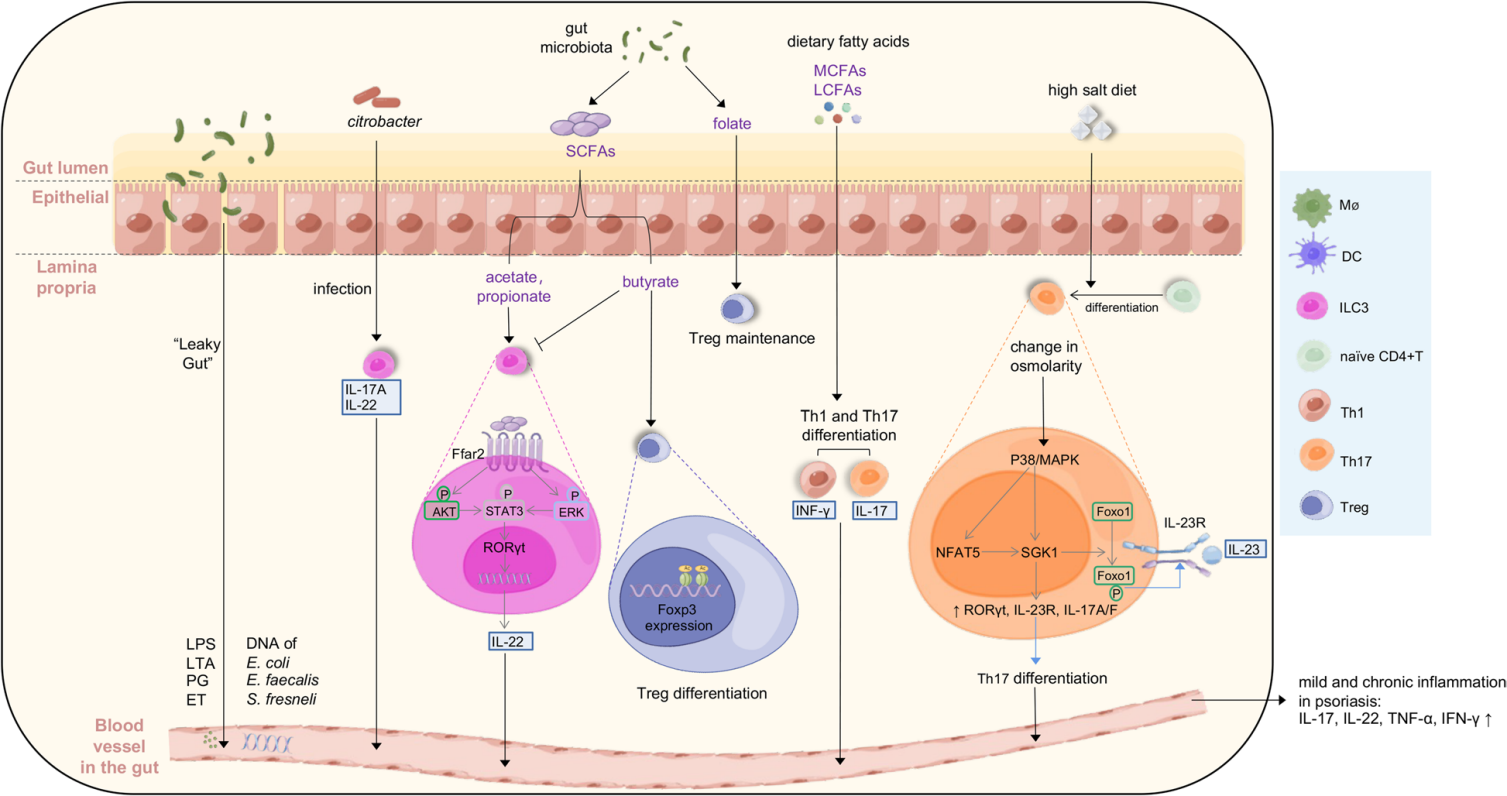
The dysbiosis of gut microbiota and diet may induce mild and chronic inflammation in psoriasis (by Figdraw, www.figdraw.com). “Leaky gut”, characterized by an increase in intestinal permeability, can induce the release of potent inflammagens such as LPS, LTA, ET, and PG, as well as the intestinal bacterial DNA translocation into blood. Additionally, C*itrobacter* infections can stimulate the production of IL-22 and IL-17A by ILC3s, thereby contributing to mucosal immunity. The function of ILC3s is also influenced by microbial metabolites SCFAs (including acetate, butyrate, and propionate). Acetate and propionate interact with the FFAR2 receptor on colonic ILC3s, resulting in the activation of AKT or ERK signaling pathways and the subsequent release of IL-22 through the STAT3 axis, while butyrate decreases the amount of ILC3s. Simultaneously, butyrate enhances the differentiation of Treg cells and folate contributes to the maintenance of Tregs, while MCFAs and LCFAs support the cell differentiation of naïve T to Th1 and Th17. The alteration in osmolarity due to a high salt diet leads to the activation of P38/MAPK pathway, subsequently upregulating downstream targets NFAT5 and SGK1, which in turn drive the expression of transcription factors RORγt, IL-23R, IL-17A, and IL-17F, leading to the differentiation of Th17. The upregulated SGK1 promotes IL-23R expression and stabilizes Th17 differentiation through Foxo1 phosphorylation

Other than the dysbiosis of the skin microbiota, the disturbed gut microbiota also influences the pathophysiology of psoriasis [19, 104] (Fig. 3). The alteration of gut microbiota in both composition and functional potentials was confirmed in patients with psoriasis compared to healthy controls [105]. Psoriasis patients had significantly disturbed gut microbiota profiles, low bacterial diversities, and distinct relative abundances of several bacterial taxa [106]. The *Firmicutes*/*Bacteroidetes* (F/B) ratio is elevated in psoriasis and positively correlates with the PASI score. Besides *Firmicutes* and *Bacteroidetes*, 16 kinds of phylotypes at the genus level also significantly differ between psoriasis patients and healthy controls [107].

Intestinal fatty acid binding protein (FABP) is a biomarker of gut barrier integrity, and its level is positively related to the severity of psoriasis [108]. The gut microbiota dysbiosis may increase intestinal permeability, also called “leaky gut”, by reducing the thickness of the mucus layers, disturbing the proliferation and metabolism of intestinal epithelial cells, and affecting the production of AMPs [109]. Additionally, the gut bacteria may escape into blood by DCs through processes between epithelial cells without affecting tight junction function, or via microfold cells overlying Peyer's patches, presenting microbial products to APCs [110]. The leaky gut facilitates the translocation of bacteria and allows the entry of exterior antigens from the intestinal lumen to the blood and lymphatic circulations, driving both local and systemic immune responses [109]. Compared to other patients and healthy control groups, the increased bacterial DNA translocation in blood samples of those suffering from plaque psoriasis was caused mostly by intestinal bacteria, including *Escherichia coli*, *Enterococcus faecalis*, and *Shigella fresneli*. Patients with bacterial DNA translocation also showed higher levels of systemic inflammatory response [111]. Another study also reported that bacterial DNA was observed in the blood of 25% of patients with plaque psoriasis, and bacterial translocation was more likely to happen among patients grouped in enterotype 2 (predominance of *Prevotella*) compared to patients classified in other enterotypes [112]. These microbes may release highly potent inflammagens such as lipopolysaccharide (LPS) and lipoteichoic acid (LTA) after being reactivated, which may contribute to the mild and chronic inflammation in the host organism, from which psoriasis patients suffer [113, 114]. Psoriasis can also be exacerbated by the bacterial endotoxins (ET) and PGs absorbed from the gut, which has been proven by the psoriasis treatment by preventing their absorption or breaking up endotoxins [115].

Moreover, the microbiota can modify immune activity through microbial metabolites in the gut. Short-chain fatty acids (SCFAs), as the major fermentation products of non-digestible carbohydrates by gut microbiome, mainly include acetate, butyrate, propionate [116]. Among them, butyrate was reported to enhance histone H3 acetylation at the promoter region of the Foxp3 locus, suggesting its potential to impact the differentiation of Treg cells [117]. Folate come from both gut microbiota and diet [118], and dietary folate has a selective effect on the maintenance of Foxp3 + Tregs [119]. As one of the host tryptophan metabolic pathways, the kynurenine routes can convert mature DCs into tolerogenic ones via indoleamine 2,3 dioxygenase, thereby enhancing Tregs and suppressing effector T cells [120]. These results may propose that microbiota metabolites act as non-infectious risk factors for psoriasis by triggering the differentiation of intestinal T cells.

Recently, much attention has focused on the function of group 3 innate lymphoid cells (ILC3s). ILC3s are primarily found in the intestine and skin [121] and are considered to play a pathogenic role in psoriasis by producing IL-17A and IL-22 [122]. When the body is infected with certain extracellular pathogens such as *Citrobacter*, ILC3s produce IL-22 and/or IL-17 for mucosal immunity against the pathogens [123]. The function of ILC3s can be also regulated by microbial metabolites such as SCFAs, including acetate, butyrate, and propionate. Butyrate can be produced by the *Firmicutes*, while acetate and propionate are mostly produced by the *Bacteroidetes* [124]. In mice, acetate and propionate bind to the SCFA receptor FFAR2 on colonic ILC3s, activate AKT or ERK signaling, and increase ILC3-derived IL-22 through an AKT and STAT3 axis [125]. However, microbiota-derived butyrate shows an opposite effect and decreases the amount of ILC3s in Peyer’s patches [126].

The treatment of probiotics has demonstrated potential benefits in the improvement of psoriasis, though no standardized treatment has been formulated [127, 128]. Fecal microbiota transplants offered another possible therapeutic strategy as they alleviate the autoimmune disease by allowing “eubiosis” from a healthy fecal microbiome to recolonize the gut of the affected patients [129]. In the future, a better understanding of microbiota dysbiosis would undoubtedly shed light on the treatments to alleviate psoriasis.

## Dysregulated lipid metabolism

**Fig. 4:**
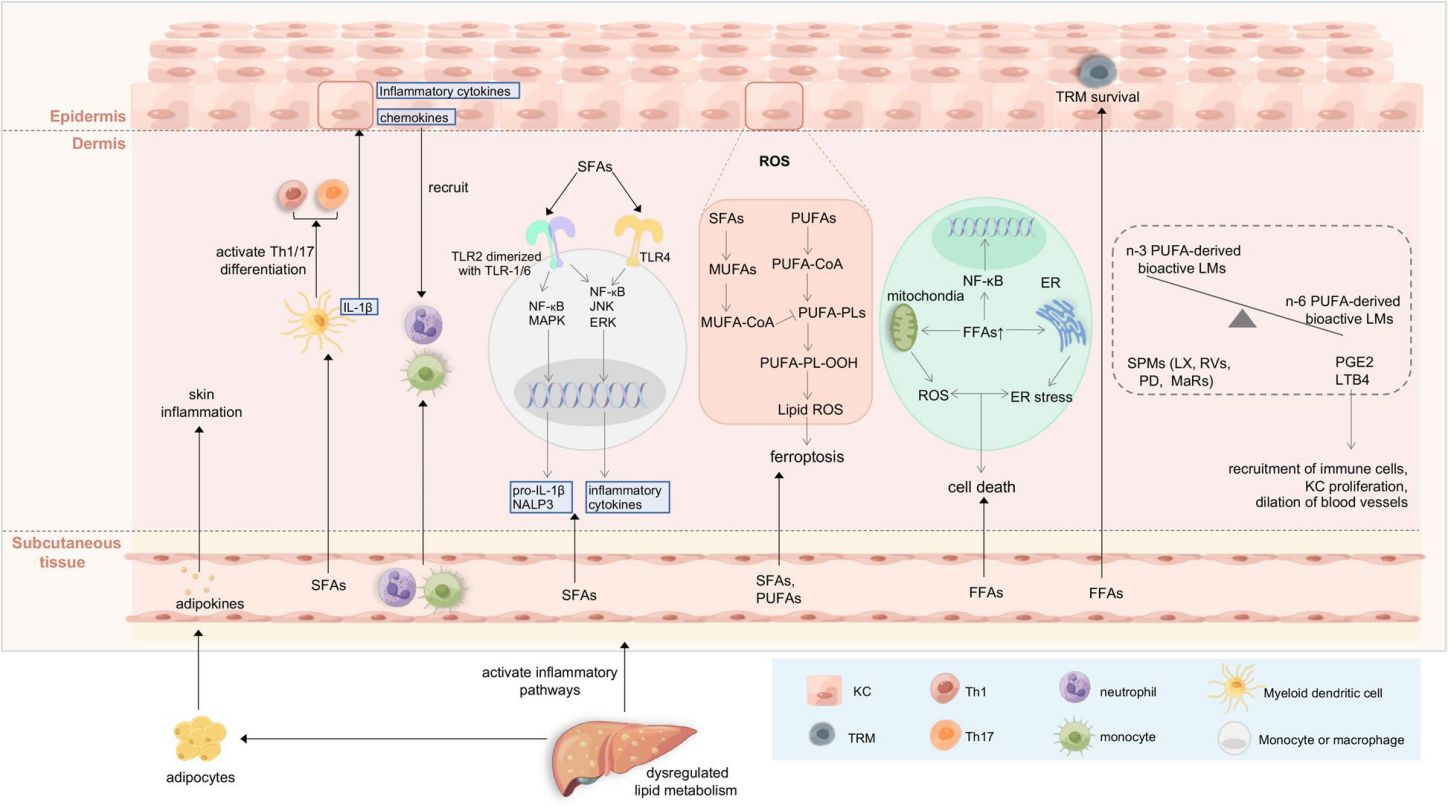
Dysregulated lipid metabolism is involved in the pathogenesis of psoriasis (by Figdraw, www.figdraw.com). Adipokines secreted by hypertrophic adipocytes modulate skin inflammation. Additionally, circulating FFAs including SFAs and PUFAs play a critical role in the development of psoriasis. SFAs are able to induce Th1/Th17 differentiation by activating DCs and also stimulate myeloid DCs to produce various proinflammatory cytokines, such as IL-1β. These proinflammatory cytokines subsequently promote the secretion of chemokines and inflammatory cytokines from KCs, leading to the recruitment of neutrophils and monocytes to the skin. Moreover, SFAs modulate the NALP3 inflammasome in monocytes or macrophages and inflammasome-mediated IL-1β secretion through the activation of TLR2 and TLR4. The lipid peroxidation of KCs in psoriasis, which initiate with the accumulation of ROS, ultimately results in KC-ferroptosis. In brief, accumulated PUFAs are catalyzed to PUFA-CoA and finally esterified into PUFA-PLs, which undergo peroxidization to form PUFA-PL-OOH. PLOOH sensitizes the cell to ferroptosis by generating lipid hydroxyl radicals and lipid peroxyl radicals. Other than ferroptosis, excess saturated FFAs in nonadipose cells can elicit both ROS and ER stress through lipid metabolism and signaling pathways, ultimately leading to the cell death. FFAs support the survival of TRM cells in the epidermis as well. The bioactive LMs derived from n-3 PUFA and n-6 PUFA exhibit contrasting anti-inflammatory and pro-inflammatory properties in psoriasis, respectively. Specifically, specialized pro-resolving lipid mediators (SPMs) derived from n-3 PUFA, including LXs, RVs, PDs, and MaRs, may resolve the psoriatic inflammatory. Conversely, n-6 PUFA-derived LMs, such as PGE2 and LTB4, contribute to neutrophil chemotaxis and KC proliferation

The association of obesity and dyslipidemia with psoriasis has been indicated by many studies [21, 130], but the molecular link between them is incompletely characterized. In general, adipose tissue may serve as an immune organ in patients with dysregulated lipid metabolism through hypertrophic adipocytes’ secretion of high amounts of hormones and cytokines (also called adipokines), including IL-6, TNF-α, and leptin, which modulate the inflammatory pathways and the activity of immune cells [131, 132]. Various psoriasis RNA-seq datasets have shown that lipid metabolism pathways are deeply involved in the pathogenesis of psoriasis [133]. Figure 4 illustrates the potential mechanisms linking dyslipidemia to psoriasis.

Recent studies have suggested that dietary components, independent of obesity-associated parameters, may play a critical role in the exacerbation of psoriasis [134–137]. A study using a mouse psoriasis model provided evidence that dietary free fatty acids (FFAs), especially saturated fatty acids (SFAs), are key amplifiers of psoriatic dermatitis. There are possible underlying mechanisms of SFAs-induced exacerbation of psoriatic dermatitis. Circulating SFAs are transported into the skin and induce the production of various proinflammatory cytokines from myeloid DCs, such as IL-1β. These proinflammatory cytokines subsequently facilitate the secretion of chemokines and inflammatory cytokines from KCs, which results in the recruitment of neutrophils and monocytes to the skin as well as the amplification of psoriatic dermatitis [134, 138]. SFAs could also modulate the NALP3 inflammasome in monocytes or macrophages and inflammasome-mediated IL-1β secretion through the activation of TLR2 and TLR4 [139, 140]. Another mechanism of SFAs in the exacerbation of psoriatic dermatitis is to promote Th1/Th17 differentiation via the activation of DCs [135, 141]. Additionally, the increase of FFAs in the body may be conducive to the recurrence of psoriasis by supporting the survival of TRM cells in the epidermis [142]. Moreover, components of dietary FFAs, middle- and long-chain fatty acids (MCFAs and LCFAs) direct the gut-shaped Th cell differentiation [143], which is opposite to the Treg differentiation by butyrate described above (Fig. 3).

Other than SFAs, polyunsaturated fatty acids (PUFAs) and PUFA-derived bioactive lipid mediators (LMs) were reported to mediate the inflammatory response in psoriasis. Among them, bioactive LMs derived from two PUFAs, linoleic acid (LA, n-6 PUFA) and α-linolenic acid (ALA, n-3 PUFA), which are respectively known for their pro- and anti-inflammatory properties in psoriasis [144]. As the representative n-6 PUFA-derived LMs, LTB4 contributes to neutrophil chemotaxis and PGE2 contributes to KC proliferation [144]. Specialized pro-resolving lipid mediators (SPMs) that derive from n-3 PUFA, including lipoxins (Lxs), resolvins (Rvs), protectins (PDs), and maresins (MaRs), have anti-inflammatory and immunomodulating functions in psoriasis [145, 146]. A research group focusses on the identification of bioactive LMs and SPMs in human psoriasis based on liquid chromatography-tandem mass spectrometry (LC–MS/MS) analyses. According to their studies, the bioactive LMs derived from n-6 PUFAs are abundant in psoriasis skin, while resolving D1 (RvD1), resolving D5 (RvD5), protectin D1 (PD1) and its double dioxygenation isomer 10S,17S-diHDHA (a.k.a. PDx), the aspirin-triggered forms of Lipoxin A4 and Lipoxin B4 (AT-LXA4 & AT-LXB4) may be the potent SPMs to resolve the inflammatory responses in the pathophysiology of psoriasis [147, 148].

Lipid metabolism is closely related to ferroptosis, and ferroptosis promotes cell death and triggers inflammation in psoriatic KCs, which involves a series of continuous events, i.e., the accumulation of reactive oxygen species (ROS) causes lipid peroxidation and further induces ferroptosis [149, 150]. During psoriasis, an enhancement of lipid peroxidation has been demonstrated by the positive correlation between lipid peroxidation and the Th22/Th17 pathway at a single-cell level [151]. KCs are also sensitive to ferroptosis in a time- and concentration-dependent manner [151]. In the lipid metabolism of KCs, accumulated PUFAs in circulation are catalyzed to the key substrate PUFA-CoA and finally esterified into PUFA-PLs, which can be peroxidized to PUFA-PL-OOH when there is bioactive iron. Subsequently, PLOOH can sensitize the cell to ferroptosis by generating lipid hydroxyl radicals and lipid peroxyl radicals. On the contrary, MUFA-CoA, the product of monounsaturated fatty acids (MUFAs) from SFAs, can reduce the available substrate for lipid peroxidation by inhibiting the peroxidization of PUFA-PLs, thus inhibiting ferroptosis. In addition, various studies have shown that Ferrostatin-1 (Fer-1), an effective inhibitor of lipid peroxidation, inhibits ferroptosis and blocks inflammatory responses in psoriasis [152].

Besides oxidative stress, lipids can also initiate endoplasmic reticulum (ER) stress, which has bidirectional effects: initial lipid-induced ER stress can be cytoprotective, but prolonged FFAs-induced ER stress might promote cell death [153]. In nonadipose cells, excess saturated FFAs induce both ROS and ER stress through lipid metabolism and signaling pathways. The following dysfunction of mitochondria and the ER are key steps leading to terminal cell death [154]. Moreover, prolonged ER stress can lead to oxidative stress and lipid-induced ROS may also trigger ER stress indirectly, though the precise mechanism is not clear yet [154].

The role of lipid autoantigen in driving dyslipidemia-related autoimmune diseases has also aroused attention [155]. Psoriatic lesions contain high levels of phospholipase A2 (PLA2), which involves in the production of neolipid skin antigens. Induced by IFN-α, the cytoplasmic PLA2 group IVD (PLA2G4D) can be released from psoriatic mast cells in the form of exosomes and transferred to neighboring CD1a-expressing Langerhans cells. Then neolipid antigens are recognized by the lipid-specific CD1a-reactive T cells, which release IL-22 and IL-17A [156]. Besides CD1a-restricted T cells, other CD1 molecules (such as CD1b and CD1c) -restricted T cells also respond to self-lipids and induce the production of cytokines. In a study, CD1b-autoreactive HJ1 T cells were directly activated by some autoantigens from accumulated phospholipids and cholesterol in skin lesions. In mice with hyperlipidemic serum, increased IL-6 production by CD1b + DCs and IL-17A secretion by HJ1 T cells were observed, indicating that the potential link between hyperlipidemia and psoriasis might lie in self-lipid-reactive T cells [157].

## Psychological stress and other mental disorders

A systematic review demonstrated a possible correlation between psychological stress and the onset, severity, and recurrence of psoriasis [20]. Patients in 31–88% of cases reported stress as a trigger for psoriasis, and a higher incidence of psoriasis occurred in subjects bearing a stressful event in the previous 12 months [158]. Another case–control study, which utilized Holmes and Rahe’s Social Readjustment Rating Scale to evaluate stress life events, drew a conclusion that stress played a significant role in the development of psoriasis, particularly in terms of recurrences and extensions [159]. However, a meta-analysis reported there was no convincing evidence of this association between stressful events and psoriasis [160]. Thus, the relationship should be prospectively scrutinized in population-based studies in the future, utilizing standardized stress instruments, as well as incorporating additional physiological and biochemical stress markers [20].

**Fig. 5:**
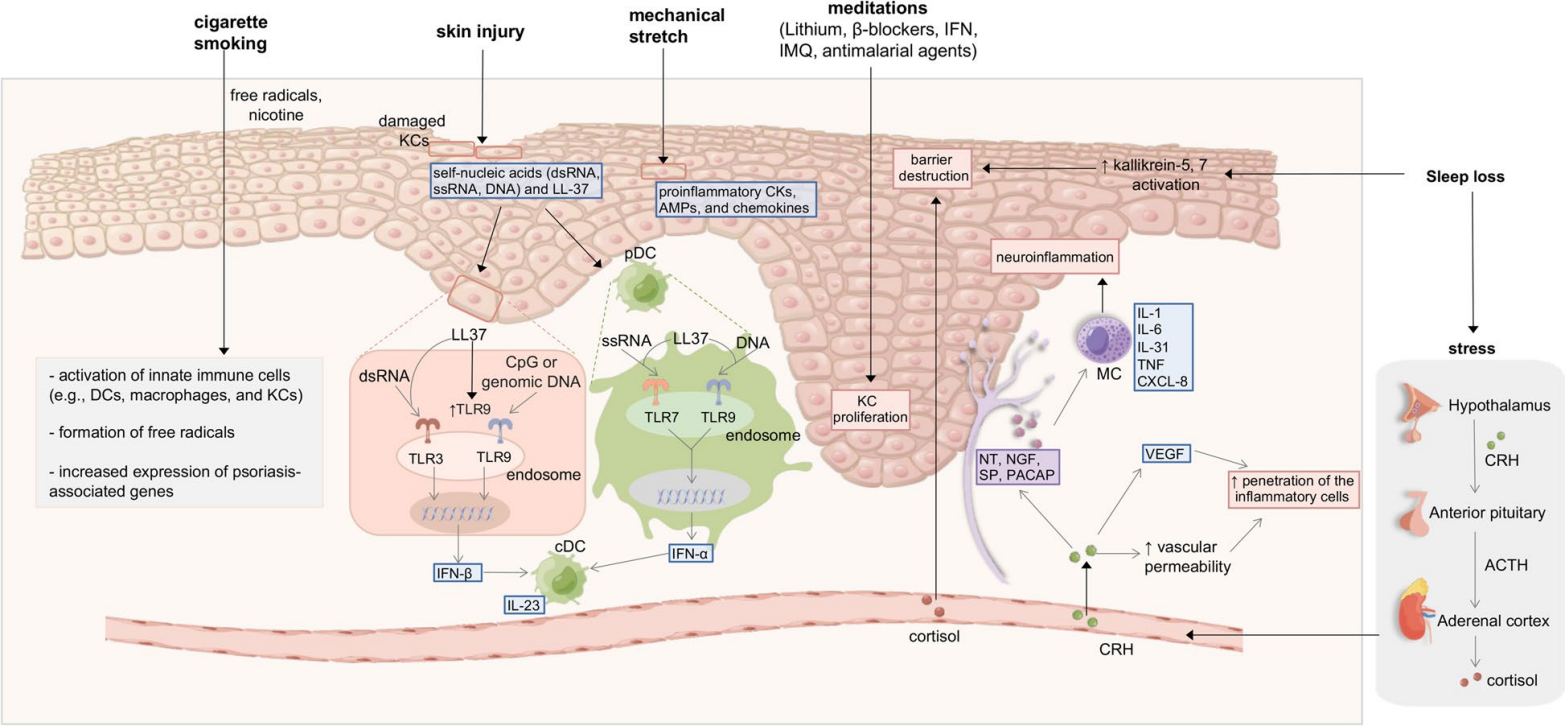
Other triggers that have been implicated in the development of psoriasis (by Figdraw, www.figdraw.com). Smoking behaviors may trigger psoriasis by activating inflammatory, oxidative, and genetic mechanisms that are mediated by free radicals and nicotine. Skin injury results in damage to KCs and subsequent release of dsRNA, ssRNA, DNA, and LL-37. pDCs mainly produce IFN-α by activating TLR7 or TLR9 with presence of DNA/ssRNA–LL-37 complexes, while KCs exposed to LL-37 produce IFN-β by recognizing CpG/genomic DNA or ssRNA–LL-37 complex via TLR9 and TLR3, respectively. Mechanical stretch induces the production of proinflammatory cytokines, AMPs, and chemokines by KCs in psoriasis. Medications, such as lithium, β-blockers, IFN, IMQ, and antimalarial agents, may trigger the proliferation of KCs. Sleep loss promotes the activities of kallikrein-5 and kallikrein-7 in the psoriatic skin, leading to epidermal barrier destruction. In addition, sleep loss also induces stress, which subsequently triggers psoriasis through the HPA axis, peripheral nervous system, and immune pathways. Upon stress, CRH activates MCs to release various cytokines and chemokines, such as IL-1, IL-6, IL-31, TNF, and CXCL-8. Stress stimulates the release of neuropeptides, including NT, SP, NGF, and PACAP from cutaneous peripheral nerve endings, thereby promoting the onset of neurogenic inflammation with MC activation. CRH also facilitates the penetration of the inflammatory cells in the psoriasis plaques by enhancing angiogenesis and increasing vascular permeability

Woźniak E et al. summarize that stress plays a role in the pathophysiology of psoriasis possibly through the hypothalamic–pituitary–adrenal (HPA) axis, immune pathways, and peripheral nervous system [161] (Fig. 5). In response to psychological stress, the hypothalamus produces corticotropin-releasing hormone (CRH), which further activates the secretion of the pituitary adrenocorticotrophic hormone (ACTH) and the adrenal cortisol. CRH is capable of suppressing the apoptosis of KCs, which is a typical phenomenon in psoriasis. On the other hand, CRH enhances angiogenesis by stimulating vascular endothelial growth factor (VEGF) and increases vascular permeability, facilitating the penetration of the inflammatory cells in the psoriasis plaques. Mast cells (MCs) can also be activated by CRH, and then release the cytokines and chemokines, including IL-1, IL-6, IL-31, TNF, and CXCL-8. Moreover, stress stimulates the release of neuropeptides from cutaneous peripheral nerve endings, leading to the development of neurogenic inflammation with the activation of MC. These neuropeptides include neurotensin (NT), substance P (SP), nerve growth factor (NGF), and the pituitary adenylate cyclase-activating polypeptide (PACAP) [161].

Apart from stress, the risk of developing psoriasis was significantly increased in patients with major depressive disorder or posttraumatic stress disorder than in the control group [162–164]. Even the association between parental common mental disorders (anxiety and depression) and offspring's risk of psoriasis has been determined [165]. Another study reported a woman with bipolar disorder subsequently developed psoriasis and experienced exacerbations in psoriatic lesions during each manic episode [166]. In accordance with the clinically elevated psoriatic inflammation in patients with autistic spectrum disorder, Nadeem et al. reported a high level of systemic inflammation in autistic mouse models, suggesting the link between autism and psoriasis activity [167]. Furthermore, previous research has established that a genetic overlap exists between severe mental disorders and psoriasis [168].

## Dysregulated sex hormones

An increasing body of research has elucidated the diverse biological and immunomodulatory effects of sex hormones on the skin. The natural course of psoriasis appears to be modulated by pregnancy, menstruation, and menopause, thereby implying a plausible involvement of female hormone-induced mechanisms in modulating skin inflammation [169, 170]. Furthermore, studies have revealed a higher prevalence and severity of psoriasis in males compared to females, especially at the estrogen abundant age, indicating distinct regulatory effects of different sex hormones on psoriasis [171].

The current consensus suggests that estrogen exerts a protective influence on psoriasis. Estrogens have been found to potentially exhibit anti-psoriatic effects by downregulating IL-1β production from neutrophils and macrophages, a process mediated through estrogen receptors α and β (ERα and Erβ) [172]. Likewise, an in vivo study demonstrated that estradiol played a protective role in imiquimod (IMQ)-induced psoriatic inflammation in mice by modulating the functions of neutrophil and macrophage [173]. In-vitro 17β-estradiol blocked the positive feedback loop of IFN-γ/interferon-induced protein of 10 kDa (IP-10), which supports Th1-mediated inflammation in psoriasis [174]. Conversely, certain studies have proposed that estrogens may possess proinflammatory properties in psoriasis, aligning with clinical observations that symptoms of psoriasis improve in some pregnant patients while worsening in others [172]. A case report has indicated that a patient undergoing tamoxifen treatment, an antiestrogenic agent, obtained remission of psoriasis symptoms, but experienced worsening symptoms during the perimenstrual cycle [175]. It is noteworthy that male psoriasis patients exhibited substantial increases in serum estradiol level compared to controls, suggesting a potential involvement of estrogen in the development of psoriasis [176]. Furthermore, an in-vivo study using an imiquimod-induced psoriasis model also indicated that estrogen plays a pro-inflammatory role in psoriasis by inducing IL-23 through Erα [177]. Collectively, these pieces of evidence support the notion that estrogen may have dual effects on psoriasis in a context-dependent manner, which leads to occasional contradictory observations [172, 178].

Existing research indicates a protective role of progestogens in psoriasis, as evidenced by the clinical observation that psoriasis often improves or resolves during pregnancy but reappears after delivery [170]. A case–control study identified an correlation between the improvement in affected body surface area and an elevation in estradiol, estriol, and the estrogen to progesterone ratio among pregnant women [179]. Some researchers have demonstrated that KCs serve as targets of progesterone by expressing progesterone receptor (PR) in psoriatic skin [180]. Furthermore, progesterone induces transcriptional alterations during pregnancy, which are enriched with genes associated with psoriasis. STAT1 and STAT3 are significantly downregulated, and their downstream targets, including IL-12β, OSM, and CXCL10, are affected [22].

A few reports have explored the role of androgen deprivation therapy (ADT) in advanced prostate cancer (PCa) as a potential exacerbating or alleviating factor for psoriasis. A case report demonstrated psoriasis exacerbation in a PCa patient following ADT [181]. Conversely, an investigation found a correlation between ADT and a decreased risk of psoriasis [182]. A separate study revealed a significant inverse correlation between total testosterone or free testosterone and PASI, irrespective of age group [183].

In summary, current investigations exploring the influence of sex hormones on psoriasis primarily rely on observational studies with a dearth of in-depth mechanistic exploration. Those somehow contradictory findings on estrogen and androgen suggest the need for additional high-quality evidence to better comprehend the intricate association between sex hormones and the pathogenesis of psoriasis.

## Other environmental triggers

The potential mechanisms responsible for triggers that cannot be classified into infectious factors, dysbiosis of skin microbiota, dysbiosis of gut microbiota or dysregulated lipid metabolism are illustrated in the Fig. 5.

### Skin trauma or pressure

Skin trauma or pressure can trigger psoriasis, known as the Koebner phenomenon (KP) [6]. Cupping therapy, as traditional Chinese medicine, was used to heal psoriasis, but it is now controversial because some psoriasis patients develop localized skin lesions through KP instead of achieving desired therapeutic results. Cupping therapy leads to KP at the cupped sites in psoriasis patients [184, 185], and Hijama (a form of wet cupping performed in Middle East countries) results in KP only in the incision areas [186]. During skin injury, damaged KCs release self-nucleic acids, including dsRNA, single-stranded RNA (ssRNA) and DNA, and induce the expression of LL-37. LL-37 enables ssRNA or DNA recognition in plasmacytoid DCs (pDCs) by TLR7 or TLR9, which finally leads to the secretion of IFN-α [187–189]. LL-37 exposure can also induce the production of IFN-β, either through a DNA-LL-37 complex–independent mechanism or through the recognition of dsRNA by TLR3. For the former mechanism, LL-37 increase TLR9 expression, thereby promoting the recognition of TLR9 ligands, such as CpG or genomic DNA [190, 191]. IFN-α from pDCs along with IFN-β from KCs promote the maturation of conventional DCs (cDCs). The recurrence of psoriasis at trauma sites has been attributed to the accumulation and reactivation of TRM cells occurring at the sites [192].

A case report described that a woman developing psoriasis vulgaris complained of new psoriasis lesions after a tissue expander insertion. Mechanical stretch was suspected to trigger ATP (Adenosine 5'-triphosphate) release from KCs and subsequent production of Th17-polarizing cytokines, like pro-IL-1β and IL-6. The epidermal Langerhans cells could also be activated by the released ATP [193]. In a murine model of skin expansion, epidermal hyperproliferation, impaired skin barrier function, along with upregulation of psoriasis-associated cytokines in epidermal KCs were observed. In human KCs, a continuous stretching regime resulted in the production of psoriasis-associated proinflammatory cytokines, AMPs, and chemokines [194]. In addition to stretch, the scratch injury to KCs triggers the KP through cytokines or chemokines CCL20 and to a less extent CXCL8 in a scratch-line-number-dependent manner [195].

### Lifestyles

The prevalence of ever smoking is higher in psoriatic patients compared with the general population [7], and the suggestive causal effect of smoking initiation and cessation on psoriasis was revealed [196]. Smoking intensity and duration may have a dose-dependent effect on the incidence of psoriasis [197, 198]. As an independent risk factor for the development of psoriasis, smoking has many negative effects on psoriasis patients, including a higher PASI score, elevated nail involvement, and the development of cardiovascular diseases [199].

Smoking may trigger psoriasis through inflammatory, oxidative, and genetic mechanisms. Nicotine stimulates innate immune cells, such as DCs, macrophages, and KCs, by releasing inflammatory cytokines. Besides, smoking initiates the formation of free radicals that activate protein signaling pathways involved in psoriasis. In the aspect of genetics, smoking upregulates the expression of psoriasis-associated genes, including HLA-C*06:02, HLA-DQA1*0201 and CYP1A1 [200]. A recent study has elucidated the involvement of CHRNA5, a nicotinic receptor gene, in the development and pathogenesis of psoriasis. Silencing CHRNA5 could inhibit the proliferation and migration of human KCs [201]. Interestingly, smoking also increases the risk of PsA in the general population, but smoking appeared a protective effect among psoriasis patients, which is known as “smoking paradox” [202]. However, a very recent Mendelian randomization study encompassing 105,912 individuals with full information on lifestyle factors, biochemistry, and genotype data suggests that smoking is an independent, but not a causal risk factor for psoriasis [203].

Sleep disorders have been commonly considered one of the risk factors for psoriasis. A nationally representative population-based dataset suggested that the risk of psoriasis and PsA increased when obstructive sleep apnea occurred [8]. Sleep loss may alter barrier homeostasis and the stratum corneum integrity through insomniac psychological stress [204]. Researchers revealed that pro-inflammatory cytokines (IL-1β, IL-6, and IL-12) were significantly increased and anti-inflammatory cytokines (e.g., IL-10) were decreased in mice with psoriasis after sleep deprivation. Sleep loss also promoted the activities of kallikrein-5 and kallikrein-7 in the psoriatic skin, which affected the epidermal barrier and led to the development of psoriasis [205]. Furthermore, cortisol increases in some sleep disorders like insomnia [206]. Cortisol stimulates skin MCs, disrupts skin barrier function, and upregulates pro-inflammatory cytokines, which further exacerbate psoriasis [207].

Dietary factors are being widely investigated for their role in psoriasis pathogenesis currently [9]. Some studies have addressed the potential role of gluten in psoriasis in several publications. Clinical improvements were seen in 73% of patients after adhering to a gluten-free diet for three months, and Ki67 lymphocytes were also reduced in the psoriatic dermis [208, 209]. Other than gluten, the increased sodium chloride (NaCl) intake is considered to have a potential effect on the pathogenesis of psoriasis (Fig. 3). Under high-salt conditions, activated p38/MAPK pathway can upregulate downstream targets nuclear factor of activated T cells 5 (NFAT5) and serum/glucocorticoid-regulated kinase 1 (SGK1). The upregulation of target genes can drive the expression of transcription factors RORγt, IL-23R, IL-17A, and IL-17F, which lead to the differentiation of psoriatic Th17 cells from naïve CD4 + T cells. SGK1 is critical for promoting IL-23R expression and stabilizing Th17 cell differentiation through the phosphorylation of Foxo1 [210, 211].

A complex and multifactorial relationship exists between psoriasis and alcohol consumption. Psoriatic patients have higher rates of excessive drinking than general people [212], and abuse of alcohol increases the severity of psoriasis and reduces treatment effectiveness [213]. There is also an increased risk of death in patients with moderate to severe psoriasis, and alcohol is a major contributing factor [214]. However, an investigation reported that alcohol consumption is not significantly linked to psoriasis development [215], and a Mendelian randomization study found no causal relationship between alcohol consumption and psoriasis as well [196]. There is still insufficient evidence to determine whether alcohol consumption implicates the onset and recurrence of psoriasis.

### Medications

Numerous medications can trigger psoriasis, such as lithium, β-blockers, antimalarial agents, nonsteroidal anti-inflammatory drugs, angiotensin-converting enzyme inhibitors, IFN, IMQ, terbinafine, statins, fibrates, and anti-programmed cell death protein 1 (PD-1) or anti-programmed death-ligand 1 (PD-L1) antibodies [10–13, 15]. In rare cases, TNF inhibitors may also paradoxically induce psoriasis [14].

Psoriasis is the most common cutaneous adverse effect of lithium [10]. The incidence of inducing and exacerbating psoriasis resulting from lithium ranges from 3.4 to 45% [216]. Lithium stimulates cell communication between psoriatic KCs and lymphocytes by inducing the release of IL-2, TGF-α, and IFN-γ [217]. Lithium also inhibits glycogen synthase kinase-3, a serine-threonine kinase, contributing to the activation of NFAT2 and the proliferation of human KCs [218]. Furthermore, lithium inhibits monophosphatase, an essential pathway for the recycling of inositol in intracellular signaling [219]. Lithium then interferes with intracellular calcium channels through the reduction of inositol, thus affecting the proliferation and differentiation of KCs [10].

When treated with β–blockers, CAMP-an intracellular messenger responsible for promoting cell differentiation and inhibiting proliferation-is shown to be decreased in the epidermis, finally leading to excessive proliferation of KCs [220]. In addition, important differences have been characterized in protein tyrosine phosphorylation activities between psoriatic T cells and controls, and the induction of protein tyrosine kinases is crucial in the activation and proliferation of cells including lymphocytes and KCs [221, 222].

Along with the rapidly growing use of anti-PD-1 or PD-L1 antibodies in the treatment of late-stage malignancies, cases of anti-PD-1/PD-L1-induced psoriasis have been gradually reported [223]. Exacerbation of existing psoriasis and newly onset psoriasis during the treatment have both been previously described [224]. Some researchers suggest that the inhibition of PD-1 promotes skin inflammation by accelerating the infiltration of epidermal CD8 + T cells, which are involved in pathogenic crosstalk with KCs. They further demonstrated the potential efficacy of IL-6–targeting therapy for anti-PD-1/PD-L1-induced psoriasis [225].

Other drugs are implicated in psoriasis through distinct mechanisms as well. For example, antimalarial drugs change the activities of enzymes, such as the modulation of transglutaminase activity, which is involved in the epidermal proliferation process [226]. IMQ, the innate TLR-7/8 ligand, can rapidly trigger or exacerbate psoriasis depending on the IL-23/IL-17 axis [227]. Nonsteroidal anti-inflammatory drugs inhibit the metabolism of arachidonic acid through the cycloxygenase pathway, contributing to the accumulation of leukotrienes, which have been postulated to exacerbate psoriasis [10].

Interestingly, psoriasis can also be triggered by biological agents, which is considered a paradoxical response. A study showed 216 reported cases of suspected TNF inhibitor-induced or -exacerbated psoriasis, which occurred more frequently with infliximab and was most prevalent in the first year of treatment for Crohn's disease and rheumatoid arthritis [228]. A retrospective analysis of patient with TNF inhibitor-induced psoriasis also yields consistent findings, indicating that infliximab is the predominant triggering agent, while Crohn's disease and rheumatoid arthritis are the most common primary conditions [229]. The paradoxical response may be associated with altered immunity induced by inhibiting TNF activity in predisposed individuals [14]. The pathogenesis is also thought to involve the IL-23/Th17 axis in the setting of TNF suppression [228].

One of the major unresolved mysteries is that psoriasis lesion often recur in the identical areas after the discontinuation of biologics targeting TNF-α, IL-23 and IL-17A/IL-17RA [230–232]. Currently, the most prevailing notion is that the existing biologics primarily serve to suppress the activities of pathogenic immune cells, rather than completely eliminating them [25].

## Conclusions

This review provides a comprehensive discussion on the risk factors and underlying pathomechanism that contribute to the onset and recurrence of psoriasis. The development of psoriasis is complicated, likely caused by multiple triggering factors rather than a singular trigger. These triggering events could occur independently under different conditions or, alternatively, they exhibit accumulative or synergistic effects. It is therefore difficult to definitively attribute the disease to specific triggers. Though *S. pyogenes* infection has been widely acknowledged as a trigger for psoriasis, supported by a substantial body of research, triggers beyond *S. pyogenes* warrant further investigation to ascertain their role in initiating psoriasis.

Given that psoriasis is triggered by the environmental risk factors on a genetic basis, the disease prevention and management deserve due attention. A guideline on the risk assessment and disease management of psoriasis could be developed according to those clear triggering factors, which is helpful for the earlier diagnosis of mild or atypical cases and the precision management of psoriasis. For example, infection history (not only *S. pyogenes* infections but also other infections listed in this review), obesity and high blood lipid levels, excessive psychological stress, smoking, sleep disorder, a high-salt diet, and a history of taking specific medications should be considered as risk factors of psoriasis. From the patient's perspective, removing these risk factors is crucial for their personal management of the disease.

From a therapeutic point of view, patients may benefit from earlier treatments targeting the “beginning”, including but not exclusively antibiotic therapy, standardized probiotic supplementation, and anti-hyperlipidemia treatment, rather than solely focusing on treatments targeting the “pretermination”, such as the use of biological agents. Understanding the role of triggers in the pathogenesis of psoriasis would also provide clues to develop new therapies that target the triggering mechanisms during the onset and recurrence of psoriasis.

## Data Availability

Data sharing is not applicable to this article as no datasets were generated or analyzed during the current study.

## Abbreviations

KCs: Keratinocytes
DCs: Dendritic cells
Th: T helper
TLR: Toll-like receptors
IFN: Interferon
TNF: Tumor necrosis factor
IL: Interleukin
GWAS: Genome-wide association studies
TRM: Tissue-resident memory T cells
K17: Keratin 17
CLA + T cells: Cutaneous lymphocyte-associated T cells
PG: Peptidoglycan
PsA: Psoriatic arthritis
APCs: Antigen-presenting cells
RIG-I: Retinoic acid inducible-gene I
HIV: Human Immunodeficiency Virus
HPV: Human papillomavirus
SARS-CoV2: Severe Acute Respiratory Syndrome Coronavirus 2
NLRP1: Nucleotide-binding domain and leucine-rich repeat pyrin domain-containing protein 1
PRRs: Pattern recognition receptors
dsRNA: Double-stranded RNA
PASI: Psoriasis Area and Severity Index
AMPs: Antimicrobial peptides
F/B: *Firmicutes*/*Bacteroidetes*
FABP: Fatty acid binding protein
LPS: Lipopolysaccharide
LTA: Lipoteichoic acid
ET: Endotoxins
SCFAs: Short-chain fatty acids
Tregs: Regulatory T cells
MCFAs and LCFAs: Middle-and long-chain fatty acids
ILC3s: Group 3 innate lymphoid cells
FFAs: Free fatty acids
SFAs: Saturated fatty acids
PUFAs: Polyunsaturated fatty acids
LMs: Lipid mediators
LA: Linoleic acid
ALA: α-Linolenic acid
LC–MS/MS: Liquid chromatography-tandem mass spectrometry
SPMs: Specialized pro-resolving lipid mediators
Lxs: Lipoxins
Rvs: Resolvins
PDs: Protectins
MaRs: Maresins
RvD1: Resolving D1
RvD5: Resolving D5
PD1: Protectin D1
AT-LXA4 & AT-LXB4: Aspirin-triggered forms of Lipoxin A4 and Lipoxin B4
ROS: Reactive oxygen species
MUFAs: Monounsaturated fatty acids
Fer-1: Ferrostatin-1
ER: Endoplasmic reticulum
PLA2: Phospholipase A2
PLA2G4D: PLA2 group IVD
HPA: Hypothalamic–pituitary–adrenal
CRH: Corticotropin-releasing hormone
ACTH: Adrenocorticotrophic hormone
VEGF: Vascular endothelial growth factor
MCs: Mast cells
NT: Neurotensin
SP: Substance P
NGF: Nerve growth factor
PACAP: Pituitary adenylate cyclase-activating polypeptide
Erα: Estrogen receptors α
Erβ: Estrogen receptors β
IMQ: Imiquimod
PR: Progesterone receptor
ADT: Androgen deprivation therapy
PCa: Prostate cancer
KP: Koebner phenomenon
ssRNA: Single-stranded RNA
pDCs: Plasmacytoid DCs
cDCs: Conventional DCs
NaCl: Sodium chloride
NFAT5: Nuclear factor of activated T cells 5
SGK1: Serum/glucocorticoid-regulated kinase 1
PD-1: Programmed cell death protein 1
PD-L1: Programmed death-ligand 1
